
JOURNAL INFORMATION
==============================
NLM Title Abbreviation: ScientificWorldJournal
Journal ID: ScientificWorldJournal
NLM Journal ID: 101131163
Journal ID: TSWJ

The Scientific World Journal

ISSN: 2356-6140
EISSN: 1537-744X
Publisher: Wiley

ARTICLE INFORMATION
==============================
PMCID: PMC6084716
PMID: 30147609
DOI: 10.1100/tsw.2001.223
Article ID: 917421
Article version: 1
Subjects: Short Report

Identification of Renox, a Novel Superoxide Producing Enzyme in Kidney

Geiszt Miklós
Leto Thomas L. *
Laboratory of Host Defenses, NIAID, National Institutes of Health, USA
*Thomas L. Leto: tleto@nih.gov
Publication date: 2001
Electronic publication date: 2001 Aug 17
Volume: 1
First page: 130
Last page: 130
Copyright: Copyright © 2001 Miklós Geiszt and Thomas L. Leto.
Copyright year: 2001
License: This is an open access article distributed under the Creative Commons Attribution License, which permits unrestricted use, distribution, and reproduction in any medium, provided the original work is properly cited.
License URL: https://creativecommons.org/licenses/by/3.0/

==============================
Miami *Nature Biotechnology* Short Reports
*TheScientificWorld* (2001) 1 (S3), 130SR
ISSN 1532-2246; DOI 10.1100/tsw.2001.223

### IDENTIFICATION OF RENOX, A NOVEL SUPEROXIDE PRODUCING ENZYME IN KIDNEY

Miklós Geiszt and Thomas L. Leto*

Laboratory of Host Defenses, NIAID, National Institutes of Health
* tleto@nih.gov

**INTRODUCTION**. Reactive oxygen species (ROS) have a crucial role in diverse physiological and pathological processes. ROS production by phagocytic cells is essential for normal host defense (1) while ROS production by nonimmune cells has important roles in apoptosis, mitogenic responses and oxygen sensing (2,3). In phagocytic cells superoxide is produced by the NADPH oxidase enzyme complex that transfers electrons from NADPH to molecular oxygen (1). The electron-transporting component of the NADPH-oxidase is $gp91^{phox}$, a glycosylated flavoprotein that forms a hetero-dimer with $p22^{phox}$.

We have identified novel $gp91^{phox}$ homolog in kidney referred to as a renal oxidase or Renox, which is highly expressed in renal tubular cells and may fulfill the function of the putative oxygen sensor in the kidney.

**METHOD**. Full-length murine Renox cDNA was isolated by 5` and 3` RACE from mouse kidney RNA as a template for cDNA synthesis. Gene expression was analyzed by Northern blot and *in situ* hybridization. Functional studies (superoxide production and proliferation) were performed by transfecting NIH 3T3 cells with the Renox cDNA.

**RESULTS**. The murine Renox is a 578 amino acid long protein showing 40% sequence identity and 57% similarity to mouse $gp91^{phox}$ while the human homolog is also a 578 amino acid long protein with 90% identity to its mouse counterpart. Renox contains conserved features considered critical for NADPH oxidase function, namely six hydrophobic segments within the amino-terminal portion, proposed as membrane-embedded domains involved in transmembrane electron transport, as well as sequence motifs corresponding to proposed binding sites for heme, flavin, and NADPH.

Northern blot experiments revealed that Renox is highly expressed in the kidney. In situ hybridization experiments on mouse kidney sections demonstrated that Renox is present in proximal tubular cells of the kidney while in human kidney inner medulary collecting duct cells show strong expression.

Renox-transfected cells showed increased superoxide production when compared with control lines and Renox-transfected fibroblasts showed drastic changes in cellular morphology and a decreased rate of proliferation.

**DISCUSSION**. Based on its expression in the proximity of erythropoietin production and its demonstrated capacity for production of superoxide we propose that Renox is a likely candidate for the oxygen sensor function in kidney.

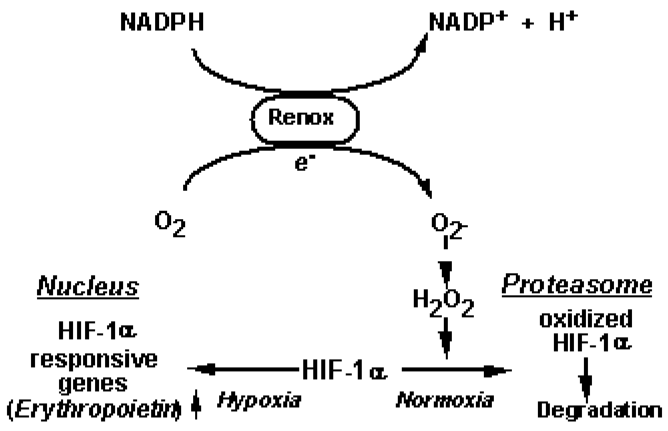

**Fig. 1.** Proposed scheme for down-regulation of erythropoietin production by Renox-generated ROS.

### REFERENCES.

1. Leto, T.L. (1999) in Inflammation Basic Principles and Clinical Correlates (Gallin, J.I. and Snyderman, R., Ed.) Lippincott Williams &Wilkins, Philadelphia, pp. 769-787
2. Ebert, B.L. and Bunn, H.F. (1999) Blood 94, 1864-1877
3. Finkel, T. and Holbrook, N.J. (2000) Nature 408, 239-247
